# The effect of different types of anemia on HbA1c levels in non-diabetics

**DOI:** 10.1186/s12902-023-01280-y

**Published:** 2023-01-28

**Authors:** Basil A. Alzahrani, Hassan K. Salamatullah, Faisal S. Alsharm, Jamil M. Baljoon, Abdullah O. Abukhodair, Mohammed Eldigire Ahmed, Hebah Malaikah, Suhaib Radi

**Affiliations:** 1 College of Medicine, King Saud Bin Abdulaziz University for Health Sciences, Jeddah, Saudi Arabia; 2 King Abdullah International Medical Research Center, Jeddah, Saudi Arabia; 3 College of Sciences and Health Professions, King Saud Bin Abdulaziz University for Health Sciences, Jeddah, Saudi Arabia; 4 Department of Pediatrics Endocrinology, Ministry of the National Guard-Health Affairs, Jeddah, Saudi Arabia; 5 Department of Internal Medicine, Division of Endocrinology, Ministry of the National Guard-Health Affairs, Jeddah, Saudi Arabia

**Keywords:** HbA1c, iron deficiency anemia, Megaloblastic anemia, β-Thalassemia trait, Sickle cell anemia, Glycated hemoglobin, anemia, Hemoglobin A1C

## Abstract

**Background:**

Diabetes mellitus is one of the most common diseases worldwide with significant morbidity and mortality. HbA1c remains one of the most important methods for diagnosis and monitoring of the disease. Since HbA1c is a reflection of the glucose attached to red blood cells, factors affecting hemoglobin and red blood cells’ half-life can influence HbA1c measurements.

**Objective:**

This study aims to evaluate the effect of different types of anemia including iron deficiency anemia, sickle cell anemia, β -thalassemia trait, and megaloblastic anemia on HbA1c levels in a tertiary hospital over the past 6 years (2016–2022).

**Method:**

This is a retrospective chart review study of 324 patients including those with one of the four types of anemia mentioned above and a control group. The control group were healthy adults with normal HbA1c and hemoglobin, who were not known to have diabetes or anemia. Patients with diabetes or prediabetes based on self-reporting or elevated fasting, random blood sugar, or 2 hours post-prandial blood glucose were excluded.

**Results:**

The mean HbA1c levels were significantly higher in sickle cell anemia at 5.83% (95% CI = 5.39–6.28) and in iron deficiency anemia at 5.75% (95% CI = 5.68–5.82) when compared to the control group at 5.32% (95% CI = 5.22–5.41). However, the mean HbA1c levels in megaloblastic anemia were 5.38% (95% CI = 5.26–5.5) and 5.45% (95% CI = 5.21–5.69) in beta thalassemia trait, which were not significantly different when compared to the control group. HbA1c significantly decreased from 5.75 to 5.44% after treatment in the iron-deficient group with a *p*-value of < 0.001. Moreover, lower hemoglobin and higher red cell distribution width correlated with higher HbA1c levels in patients with sickle cell anemia.

**Conclusion:**

This study found a significant increase in HbA1c levels in iron deficiency anemia and sickle cell disease in patients not known to have diabetes. However, there was no significant effect in those patients with β-thalassemia trait and megaloblastic anemia. Treatment of iron deficiency anemia significantly decreased the HbA1c level, bringing it back to normal.

## Introduction

Diabetes mellitus is one of the most common diseases worldwide and is rapidly increasing. It is also one of the major causes of morbidity and mortality. Complications from diabetes have been correlated to the control of the disease. Despite newer targets that reflect diabetes control, like the time in range, glycated hemoglobin (i.e., hemoglobin A1C) remains the main measure of disease control. The hemoglobin A1C (HbA1c) test is used to measure an individual’s glycemic index in the last 3 months [1]. Many specialists apply this test to evaluate blood glucose levels [2]. Normal HbA1c is < 5.7%, levels between 5.7–6.4% are considered prediabetes, and a level of > 6.5% is diagnostic for diabetes [3]. Since HbA1c measures the sugar that is linked to a form of hemoglobin, abnormalities in hemoglobin can theoretically falsely affect the HbA1c level [4]. Hemoglobinopathies involve all genetic hemoglobin conditions, and they are divided into two categories, abnormal hemoglobin and thalassemia disorder. All of them happen because of deletions and/or mutations in the α- or β-globin genes [5].

Anemia is a major public health issue in many regions of the world, affecting over 30% of the world’s population. Iron deficiency anemia (IDA) is a condition in which iron is gradually depleted due to inadequate dietary intake, hemorrhage, or insufficient intestinal iron absorption. As a result, the production of iron-containing proteins such as hemoglobin (Hb) is hampered [6]. More than 2 billion individuals worldwide suffer from IDA, and it is still the leading cause of anemia [7]. A previous study discovered a link between IDA and HbA1c levels and attempted to explain the change in HbA1c levels in IDA using both hemoglobin structure modifications and HbA1c levels in old and new red blood cells [8]. The literature is divided on the effect of IDA on HbA1c levels, with some studies showing an increase in HbA1c and others showing that HbA1c was significantly lower in IDA patients compared to control groups [1, 9].

Sickle cell anemia (SCA), which is an autosomal recessive condition characterized by hemoglobin S (Hb S) production, is one of the most prevalent hereditary hemoglobinopathy diseases worldwide [10, 11]. In the United States, SCA affects about 72,000 people and approximately 2 million are carriers [11]. Hemoglobin S changes the normal shape of the red blood cells (RBCs) to become sickle-shaped cells [10]. Sickle cell disease (SCD) may alter HbA1c’s ability to accurately reflect past glycemia. When compared to people with normal hemoglobin, the lifespan of red blood cells in people with SCD may be shortened, leaving less time for glycation, which can falsely lower HbA1c levels [12].

Thalassemia is a diverse set of inherited autosomal recessive disorders, which are caused by a reduction in alpha or beta chains that compose the hemoglobin [13]. Thalassemia is classified according to the affected hemoglobin molecule into alpha- and beta-thalassemia. Beta-thalassemia is caused by the deletion of the beta-globin gene on chromosome 11, which results in the formation of fewer or no beta-globin chains [13]. It is characterized by a lack of erythropoiesis and persistent hemolysis. Heterozygous thalassemia (also known as thalassemia minor) is thalassemia that affects 1–5% of people worldwide, according to the World Health Organization. Because β-thalassemia carriers have a shorter erythrocyte lifetime, ineffective erythropoiesis and peripheral hemolysis may lower HbA1c levels [14].

Vitamin B12 deficiency is a known cause of megaloblastic anemia. Vitamin B12 is crucial for DNA synthesis during erythropoiesis, and its deficiency, which results from inadequate intake or malabsorption, is associated with the decreased synthesis of healthy, mature RBCs [15]. According to the hypothesis that HbA1c increases with aged RBCs and decreases with increased amounts of immature blood cells, HbA1c was hypothesized to be normal in megaloblastic anemia (MA) since it is associated with decreased RBC synthesis with no aged RBCs [16].

Since HbA1c measures the sugar attached to hemoglobin, abnormalities in hemoglobin can theoretically falsely affect the level of HbA1c, which can be reflected in diabetes diagnosis and monitoring. Falsely low HbA1c can delay diabetes diagnosis or give the physician the impression that diabetes is well-controlled. On the contrary, a falsely elevated HbA1c can mislabel patients with diabetes and lead to associated psychological stress and unnecessary treatment. Our study aims to evaluate the effect of different types of anemias, including IDA, SCA, β-thalassemia trait (BT), and megaloblastic anemia, on HbA1c levels in King Abdulaziz Medical City, a tertiary university hospital in Jeddah, Saudi Arabia, over the past 6 years.

## Methods

This is a retrospective chart review study, conducted on patients followed in King Abdulaziz Medical City-Jeddah from 2016 to 2022. The study was approved by the Institutional Review Board (IRB) of King Abdullah International Medical Research Center (KAIMRC). The main objective was to measure the effect of four different types of anemias on hemoglobin A1C levels. The four types of anemia were: 1. iron deficiency anemia, 2. sickle cell disease; 3. β-thalassemia trait; and 4. megaloblastic anemia. A control group consisting of patients who do not have anemia was enrolled in this study for comparison.

The inclusion criteria were patients aged 18 years or older who were diagnosed with IDA, SCA, β-thalassemia trait, or vitamin B12 related–megaloblastic anemia. We also included a control group who were healthy adults aged 18 years or older who had the HbA1c test done for screening purposes. The control group had to have normal hemoglobin and blood sugars and were not labeled as having any kind of anemia, diabetes, or prediabetes. The exclusion criteria included patients who had anemia from causes other than IDA, SCA, β-thalassemia trait, or megaloblastic anemia; a recent blood transfusion (within the past month); patients younger than 18 years of age; recent blood loss, or surgery within the past 3 months; patients with uncontrolled hypothyroidism; patients with an abnormal renal function test or abnormal liver test; and pregnant women.

To ensure that the patients in the study were not diabetic, we included only those with normal fasting blood sugar, normal random blood sugar, or a normal oral glucose tolerance test, if available. If any of these tests were abnormal, then the patient was excluded.

### Definitions

Sickle cell anemia was defined as an HbS level of 75–95%, HbF of 2–25%, and positive genetic testing. We included only those with sickle cell disease rather than the trait. Iron deficiency anemia was diagnosed based on a hemoglobin level of less than 130 g/L (13 g/dL) in men and less than 120 g/L (12 g/dL) in non-pregnant women in addition to an MCV level of less than 80 fL and a ferritin level of less than 30 mcg/L. Megaloblastic anemia is defined as a low hemoglobin level as mentioned above with a vitamin B12 level of less than 148 pmol/L. Although megaloblastic anemia can also be caused by folate deficiency, we only included those caused by vitamin B12 deficiency. Beta-thalassemia trait was diagnosed based on genetic testing and HbA2 of 4% or more. Abnormal fasting blood glucose was defined as more than 5.6 mmol/L (100 mg/dL) and an abnormal glucose tolerance test with blood sugar at more than 7.8 mmol/L (140 mg/dL). Those patients were excluded.

The patient’s data were collected from the electronic medical records and entered into the data collection sheet, which contained multiple variables: age; gender; type of anemia; comorbidities age; hemoglobin level; hemoglobin A1C; red blood cell count; mean corpuscular volume (MCV); red cell distribution width (RDW); mean corpuscular hemoglobin (MCH); creatinine level; bilirubin level; ferritin level for IDA patients; and vitamin B12 for patients with megaloblastic anemia. HbA1c is measured in our center using high-performance liquid chromatography utilizing the D-100 system from Bio-Rad.

### Sample size and data analysis

Based on sample size calculation, considering a margin of error of 5% and a confidence interval of 95%, using the Epitools sample size online calculator for case control studies, and taking the odds ratio from previous relevant studies to be 3.9, the sample size for each group of patients should be 103 with a total sample size of 412. The iron deficiency anemia group was the only group that met these numbers, along with the control group. For the remaining three groups, we included all patients with the respective type of anemia that met the inclusion criteria beginning in 2016, the year an electronic medical records system was established in our institution. It was determined that the control group matched the highest group, which was the iron deficiency anemia group.

Patients’ records were divided between data collectors. After completing the data collection process, an independent review of the correctness of the data was done by another author and then by the corresponding author. Data were entered into an Excel spreadsheet, and analysis was conducted using John’s Macintosh Project (JMP) pro 15.2.0 statistical software. Missing data and outlier values were detected and treated prior to statistical analysis. The descriptive categorical variables were presented in frequencies and percentages. The dependent factor of this study is the five groups which were IDA, SCA, β-thalassemia, megaloblastic anemia, and the control group. In addition, the descriptive numerical variables were depicted by mean and standard deviation.

Regarding the inferential statistics, chi-square, one-way ANOVA, and an unpaired T-test were utilized. A *p*-value of less than or equal to 0.05 was considered significant.

## Results

### Demographic

A total of 324 patients were included in this study. One hundred and three (31.8%) patients were diagnosed with iron deficiency anemia, 67 (20.6%) with megaloblastic anemia, 33 (10.2%) with sickle cell anemia, 17 (5.2%) with β-thalassemia trait, and 104 (32%) were included in the control group. Among the 324 patients, 194 (60%) were female, 75 (23.08%) had hypertension, 49 (15.08%) were diagnosed with dyslipidemia, 45 (13.93%) had at least one autoimmune disease, and 18 (5.56%) had ischemic heart disease. Furthermore, the mean age was 46.62 years, and the mean body-mass index (BMI) was 29.31. Of the 221 patients diagnosed with one of the four causes of anemia, only 89 (40.27%) were receiving treatment.

There was a clear female predominance in most groups of anemia. The group with the most female patients was β -thalassemia trait, in which 15 out of 17 (88.24%) were female. Moreover, 51 out of 67 (76.12%) megaloblastic anemia patients were female, as were 23 out of 33 (69.7%) SCA patients, 53 out of 103 (52.4%) IDA patients, and 52 out of 104 (50%) members of the control group. There was also a statically significant age difference between groups. For example, the group with the highest mean age was the IDA group, with a mean age of 50.78 (95% CI = 47.66–53.9). In contrast, the group with the lowest mean age was the SCA group, which had a mean age of 35.33 (95% CI = 29.8–40.87). Baseline characteristics are shown in Table 1.

**Table 1:** Baseline characteristics of participants and lab indicators before treatment by group

Variable	All *N* = 324	IDA *N* = 103	SCA *N* = 33	BT *N* = 17	Megaloblastic *N* = 67	Control *N* = 104
Gender						
Female^*^	195 (60.0)	53 (52.4)	23 (69.7)	15 (88.2)	51 (76.1)	52 (50.0)
Male	130 (40.0)	50 (47.6)	10 (30.3)	2 (11.8)	16 (23.9)	52 (50.0)
Age^*^	46.6 ± 16.7	51.0 ± 17.3	35.3 ± 11.1	39.8 ± 8.5	46.5 ± 17.5	47.2 ± 16.1
BMI	29.3 ± 7.0	29.0 ± 6.3	28.2 ± 7.0	28.5 ± 4.2	31.0 ± 9.3	29.0 ± 6.1
Hb in g/dL	N/A	9.7 ± 1.5	10.6 ± 2.0	11.1 ± 1.6	11.3 ± 2.1	13.9 ± 1.4
RBC × 10^12^/L	N/A	4.3 ± 0.60	3.9 ± 0.89	4.8 ± 1.1	4.0 ± 0.81	4.8 ± 0.51
MCV fL	N/A	75.6 ± 9.1	84.5 ± 12.4	66.4 ± 7.7	89.4 ± 9.5	88.9 ± 5.1
RDW %	N/A	16.5 ± 3.3	17.5 ± 3.8	15.1 ± 2.8	15.0 ± 4.2	13.3 ± 1.3
MCH	N/A	25.5 ± 10.5	27.3 ± 4.6	20.6 ± 2.9	28.9 ± 3.6	29.0 ± 1.9
Glu F in mmol/L^a^	N/A	5.2 ± 0.53	5.21 ± 0.5	5.07 ± 0.65	5.06 ± 0.68	5.2 ± 0.57
Creatinine in micromol/L^b^	N/A	73.2 ± 25.0	67.49 ± 31.44	60.54 ± 10.13	68.32 ± 17.98	70.12 ± 12.05
Bilirubin in micromol/L^c^	N/A	9.4 ± 6.85	23.65 ± 21.41	14.46 ± 5.24	10.94 ± 7.49	10.33 ± 5.03
Ferritin in mcg/L	N/A	22.3 ± 52.1	76.46 ± 89.51	69.86 ± 85.61	89.88 ± 153.63	89.21 ± 73.79
B12 in pmol/L	N/A	N/A	N/A	N/A	108.72 ± 23.54	N/A

*IDA* Iron Deficiency Anemia, *SCA* Sickle Cell Anemia, *BT* β-Thalassemia trait, *BMI* Body Mass Index, *Hb* Hemoglobin, *MCV* Mean Corpuscular Volume, *RDW* Red cell Distribution Width, *MCH* Mean Corpuscular Hemoglobin, *Glu F* Fasting Glucose

^*^Variables with *p*-value < 0.05

^a^To convert glucose from mmol/L to mg/dL, multiply by 18

^b^To convert creatinine from micromol/L to mg/dL, divide by 88.4

^c^To convert bilirubin from micromol/L to mg/dL, divide by 17.1

### Lab parameters

Mean hemoglobin levels before treatment were 10.6 g/dL (95% CI = 9.85–11.3) in SCA, 11.32 g/dL (95% CI = 10.82–11.83) in megaloblastic anemia, 9.67 g/dL (95% CI = 9.39–9.96) in IDA, 11.1 g/dL (95% CI = 10.26–11.87) in BT, and 13.9 g/dL (95% CI = 13.61–14.16) in the control group. RBC count was markedly lower in the SCA and megaloblastic anemia groups. In addition, as expected, MCV and MCH values were much lower in the IDA and BT groups as illustrated in Table 1. The highest mean of fasting glucose was 5.21 mmol/L (95% CI = 4.95–5.47) in SCA and the lowest was 5.06 mmol/L (95% CI = 4.82–5.3) in megaloblastic anemia. The mean ferritin level among the IDA group was 22.3 mcg/L (95% CI = 12.3–74.4), and the mean vitamin B12 level among the megaloblastic anemia group was 108.72 pmol/L (95% CI = 102.97–114.46).

### HbA1c interpretations

**Fig. 1:**
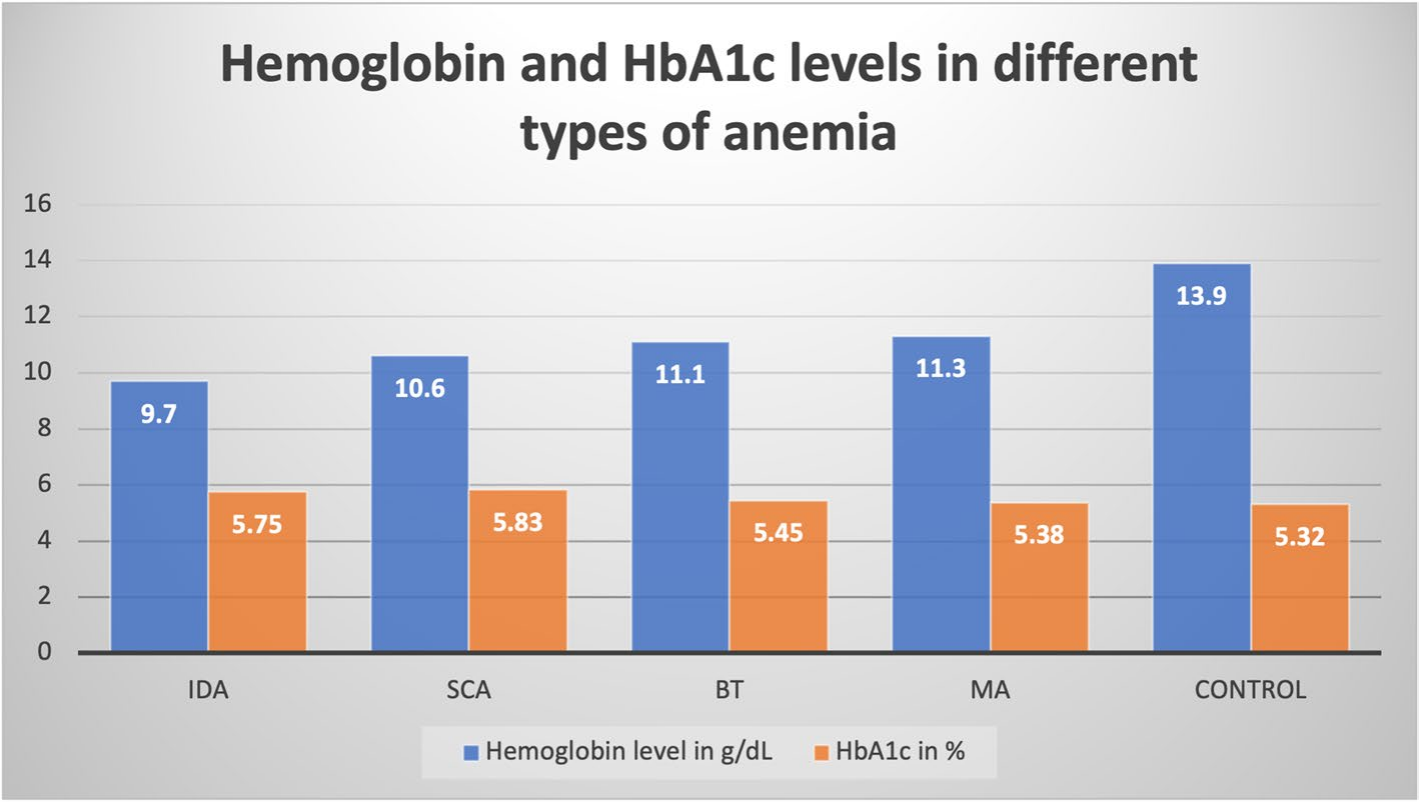
Difference between Hemoglobin levels and HbA1c levels in the different types of anemia compared to the control group. IDA: iron deficiency anemia; SCA: sickle cell anemia; BT: Beta-thalassemia trait; MA: megaloblastic anemia; HbA1c: glycated hemoglobin

Values of HbA1c before treatment of anemia were assessed for all anemic groups and compared to the control group. The mean HbA1c levels were significantly higher in SCA at 5.82% (95% CI = 5.39–6.28) and IDA at 5.75% (95% CI = 5.68–5.82) when compared to the control group at 5.31% (95% CI = 5.22–5.41). On the other hand, the mean HbA1c levels in megaloblastic anemia at 5.38% (95% CI = 5.26–5.5) and in beta thalassemia trait at 5.45% (95% CI = 5.21–5.69) were not significantly different when compared to the control group as illustrated in Table 2 and Fig. 1. In addition, the HbA1c values of the IDA group were compared before and after treatment to evaluate the effect of the hemoglobin level correction on the HbA1c value. A significant decrease in HbA1c from 5.75 to 5.44% was noticed after treatment of IDA with a *p*-value of < 0.001, as depicted in Table 3.

**Table 2:** HbA1c percentage before treatment for each group versus control group (5.32 ± 0.48)

Group	Mean ± SD	95% Confidence Interval	*P* value	Difference from Control	95% Confidence Interval
IDA	5.75 ± 0.35	5.68–5.82	0.001	+ 0.43	0.32–0.54
SCA	5.83 ± 1.26	5.39–6.28	0.001	+ 0.51	0.07–0.95
β-Thalassemia trait	5.45 ± 0.47	5.21–5.69	0.290	+ 0.13	- 0.12 – 0.38
Megaloblastic anemia	5.38 ± 0.49	5.26–5.5	0.378	+ 0.06	- 0.09 – 0.21

*IDA* Iron Deficiency Anemia, *SCA* Sickle Cell Anemia

**Table 3:** HbA1c percentage before and after treatment in IDA group

HbA1c	HbA1c Before Treatment	HbA1c After Treatment	Significance of Difference
Mean	5.75	5.44	*P* < 0.001
SD	0.41	0.52	
95% CI	5.61–5.89	5.25–5.61	

*IDA* Iron Deficiency Anemia

Furthermore, to identify whether the severity of anemia can influence HbA1c levels, hemoglobin levels of all anemic groups were divided into two groups: group 1 (Hb ≤ 9.5) and group 2 (Hb < 9.5). SCA was the only group that had a significant difference in HbA1c values between group 1, with 6.44% (95% CI = 5.19–7.68), and group 2, with 5.54% (95% CI = 5.22–5.85) and a *p*-value of 0.026. The remaining groups had no significant difference in HbA1c levels at different hemoglobin levels, even though HbA1c levels were numerically higher at lower hemoglobin levels as shown in Table 4. In the SCA cohort, the low Hb group had a significantly higher red cell distribution width (RDW) compared to the high Hb group (21.03% vs 15.74%, p-value < 0.0001). The reference range for RDW in our center is 11.5–14.5%.

**Table 4:** HbA1c level before treatment by Hb level (low and high) for each group

Group	Hb Low group (Hb ≤ 9.5)		Hb High group (Hb < 9.5)		*P* value
	Sample size	HbA1c %	Sample size	HbA1c %	
IDA	48/103	5.81 ± 0.27	55/103	5.69 ± 0.39	0.053
SCA	11/33	6.43 ± 1.85	22/33	5.53 ± 0.71	0.026
β-Thalassemia trait	3/17	5.70 ± 0.65	14/17	5.39 ± 0.42	0.150
Megaloblastic anemia	11/67	5.41 ± 0.65	56/67	5.37 ± 0.46	0.428

*IDA* Iron Deficiency Anemia, *SCA* Sickle Cell Anemia, *Hb* Hemoglobin, *HbA1c* Hemoglobin A1c

## Discussion

The HbA1c test is used to measure the amount of glycosylated hemoglobin in the blood in the past 3 months. It is used primarily to diagnose patients with diabetes mellitus and to monitor those diagnosed to assess their response to therapy. According to the American Diabetes Association, diabetes is diagnosed when HbA1c is > = 6.5%. Levels between 5.7–6.4% are considered prediabetes, while normal HbA1c is < 5.7% [3]. Conditions such as different types of anemia can affect the levels of HbA1c in the blood. In general, conditions associated with decreased erythropoiesis (e.g., IDA) can falsely increase HbA1c because of the longer half-life of red blood cells. On the contrary, increased erythropoiesis resulting in rapid turnover of red blood cells can falsely lower HbA1c. Many previous studies done in this field showed highly contradictory results. Therefore, this study aimed to measure the effects of four groups of anemia, IDA, SCA, β-thalassemia trait, and megaloblastic anemia, on HbA1c levels compared to a control group. It was found that there is a statistically significant increase in HbA1c levels in IDA and SCA patients when compared to the control group.

Regarding the IDA group, the mean HbA1c was significantly higher than the control group (5.75% compared to 5.32%). In IDA, red cell production decreases, resulting in an increased age of circulating red blood cells. This will ultimately lead to higher HbA1c levels [1]. We also demonstrated that treating anemia in iron-deficient patients significantly lowered the HbA1c to 5.44%. This finding was supported by multiple previous studies in the literature. Brooks et al. found that iron-deficient nondiabetic patients had increased HbA1c values and returned to normal after iron replacement [17]. Moreover, Gram-Hansen et al. found that normal HbA1c concentrations in iron deficiency decreased to below-normal levels after iron therapy [16]. On the other hand, Heyningen and Dalton showed no differences in HbA1c concentrations in nondiabetic patients with IDA before and after iron replacement. Their study, however, contained only 14 patients, which might be an underrepresentation [18]. El-Agouza et al. explained the HbA1c production mechanism, stating that HbA1c levels reflect a balanced relationship between HbA1c and serum glucose concentrations. The HbA1c level is expressed as a percentage of total HbA. As a result, serum glucose is allowed to remain at a steady state, despite the decrease in hemoglobin concentration, which may result in an increase in the glycated fraction [19]. In our study, patients were subdivided into two groups based on their hemoglobin level; the first group had a hemoglobin level of ≤9.5 g/dl and the second group had a hemoglobin level of > 9.5 g/dl. Although the lower hemoglobin group had a numerically higher HbA1c, this did not reach statistical significance. Ford et al. demonstrated that there is an inverse correlation between hemoglobin concentration and HbA1c levels, yet these findings were only for patients who had hemoglobin levels higher than 10 g/dl [20].

Theoretically speaking, sickle cells would have a much shorter life span compared to healthy red blood cells. This shorter life span will result in falsely lower HbA1c when compared to healthy individuals [10]. It is hypothesized that the presence of hemoglobin S results in the shorter lifespan of red blood cells and less available time for hemoglobin glycation [12]. Another proposed mechanism by which sickle cell disease can affect HbA1c is assay interference caused by hemoglobin S on some of the HbA1c measurement techniques [12]. This has been corroborated in the literature. In a study done by Lacy et al. in adults with sickle cell trait, HbA1c levels were lower among people with sickle cell trait as compared to those without it [12]. Moreover, Atabani et al. showed similar results in patients with sickle cell anemia. They measured HbA1c for sickle cell anemia and sickle cell trait patients and compared the results with a control group. Their study demonstrated that the mean HbA1c in SCA patients was 4.9%, which was significantly lower than in the control groups [21]. However, these results contradict our results.

In our study, the mean HbA1c in SCA patients was significantly higher than in the control group (5.83% vs. 5.32%), with a difference of + 0.51 (CI = 0.07–0.95). There are a few caveats that can explain these results. First, only 13 out of the 33 patients with sickle cell anemia had normal ferritin. However, even after analyzing only those with normal ferritin, the mean HbA1c was still statistically higher in the sickle cell patients compared to the control group (6.15% vs. 5.32%). Second, we included only patients with sickle cell disease, unlike some of the other studies mentioned, which included sickle cell trait patients. Third, we had a patient in the sickle cell anemia group who had an A1C of 10.6% despite not being diabetic and with normal fasting and random blood sugar results obtained multiple times. This very high A1C could have skewed the mean HbA1c. We did a secondary analysis after excluding this patient from the sickle cell disease group and the mean HbA1c was still statically higher than in the control group.

Fourth, abnormal hemoglobin, such as hemoglobin S and F, can sometimes cause an erroneous HbA1c reading. In our lab, we use high-performance liquid chromatography to measure HbA1c utilizing the D-100 system from Bio-Rad. Although this method has not been reported in the literature as being affected by abnormal hemoglobin, we think that it is still a plausible explanation for the falsely higher HbA1c in our sickle cell group [22]. Another possible explanation is the interesting finding that RDW, a measure of difference in size and volume of red blood cells, was significantly higher in the lower Hb group of patients with SCA. Bao et al. found that RDW was independently related to higher HbA1c through non-glycemic ways in patients without diabetes while it didn’t affect fasting and random blood sugar readings [23]. Finally, the number of patients with sickle cell anemia in our study is relatively small compared to those in the other studies mentioned.

There is a paucity of studies regarding the effect of β-thalassemia trait on HbA1c in non-diabetics. It is hypothesized that β-thalassemia trait can falsely lower HbA1c through multiple mechanisms. First, ineffective erythropoiesis and peripheral hemolysis can shorten the lifespan of erythrocytes, resulting in lower HbA1c. Additionally, elevated hemoglobin F levels can affect some of the laboratory methods used to measure HbA1c [14]. Our results showed no effect on HbA1c in patients with β-thalassemia trait compared to the control group. Also, there was no relation between the severity of anemia and HbA1c levels. A study by Tsilingiris et al. found that the mean HbA1c percentage in those with heterozygous thalassemia was similar to those without β-thalassemia trait. However, they demonstrated that there is an inverse relation between hemoglobin concentration and HbA1c. They concluded that this correlation is of negligible clinical significance, and they advocated for continuing to utilize HbA1c in diagnosing diabetes mellitus in those with β-thalassemia trait [14].

Megaloblastic anemia is also associated with ineffective erythropoiesis and peripheral hemolysis. This is caused by apoptosis of hematopoietic cell precursors, which results from DNA synthesis abnormalities. Both vitamin B12 and folate deficiency can cause defective DNA synthesis, but we included only patients with vitamin B12 deficiency [24]. In the group with megaloblastic anemia, the mean HbA1c was 5.38%, which showed no significant difference in comparison to the control group (5.32%). Similar to what was reported by Gram-Hansen et al., the median HbA1c level before treatment was 5.1%, which also exhibited an insignificant difference compared to the control group [16]. This supported the hypothesis that megaloblastic anemia is a type of anemia characterized by decreased RBC synthesis and no excess formation of young blood cells; thus, there will be little effect on HbA1c [25]. Pilla et al. also studied 100 patients with megaloblastic anemia before and after correcting their anemia. The baseline HbA1c was significantly higher in those with megaloblastic anemia compared to the control group, and correcting the anemia did decrease the HbA1c level significantly to the range of the control group [26]. It is worth mentioning that in their study, the hemoglobin level was significantly lower than in ours (6.1 g/dL vs. 11.3 g/dL), which could explain the different findings.

There was a female predominance in most types of anemia, 60% for the whole cohort versus 40% for males. The high prevalence of IDA and megaloblastic anemia among female patients corresponds to other studies. Sinha et al. showed female predominance among patients diagnosed with IDA [4]. This may be explained by heavy menstrual bleeding, lactation, and pregnancy [27]. Our results showed that 76.1% of megaloblastic anemia patients were female. Similarly, Green et al. stated that there is a high prevalence of this condition in female patients compared to male patients by 3.3 to 2.4%, respectively, according to the National Health and Nutrition Examination Survey (NHANES). Possible explanations include higher rates of autoimmune diseases among females, and that during pregnancy, there are multiple mechanisms that increase vitamin B12 absorption for the fetus [15]. The very-high female predominance in β-thalassemia and sickle cell anemia was surprising. Unfortunately, we could not find any local studies to support this finding and it seems that there is no gender preference in these diseases.

Our study has many limitations. First, the retrospective nature of the study renders it subject to different types of bias. Also, the sample size of the sickle cell group and β-thalassemia group was relatively small, making interpretation of findings in these two groups difficult. Some patients came to our center already undergoing some treatment for their anemia (IDA and megaloblastic) so their initial hemoglobin levels might have been lower, which can affect the results. Also, since this was a retrospective chart review study, there was a large variation in the treatment of anemia in terms of dosing and route of administration. This might have affected some of the results; however, using the post-treatment hemoglobin level was our objective way of assessing response to therapy. Moreover, the degree of iron deficiency and vitamin B12 deficiency was not severe. It would be interesting to see if severe deficiency can alter the HbA1c levels even more. Although we excluded patients with elevated fasting and post-prandial blood glucose, isolated HbA1c might still be a sign of impaired glucose tolerance and it would not be possible to differentiate this from the effect of anemia or abnormal hemoglobin. Finally, we only included patients who are not diabetics, and our results cannot be generalized to those known to have diabetes.

In conclusion, IDA and sickle cell disease can falsely increase HbA1c and correcting the anemia in IDA patients can decrease the HbA1c level back to baseline. Our study found that the level of hemoglobin was inversely related to the HbA1c level in patients with sickle cell anemia, while high RDW correlated with high HbA1c levels. Clinicians should use caution when depending on HbA1c to diagnose diabetes in those with IDA and sickle cell disease. We recommend correcting the anemia in patients with IDA before using HbA1c for diagnostic purposes. Further studies on a larger scale are needed to evaluate the effect of sickle cell disease and RDW on HbA1c.

## Data Availability

The raw data supporting the conclusions of this article will be made available by the authors, without undue reservation. Please contact the corresponding author, Suhaib Radi, to request any data or supporting files.

## Abbreviations

BMI: Body Mass Index
BT: β-Thalassemia trait
Glu F: Fasting Glucose
Hb: Hemoglobin
HbA1c: Hemoglobin A1c = glycated hemoglobin
HbF: Fetal hemoglobin
HbS: Hemoglobin S
IDA: Iron Deficiency Anemia
MA: Megaloblastic anemia
MCH: Mean Corpuscular Hemoglobin
MCV: Mean Corpuscular Volume
RBC: Red blood cells
RDW: Red cell Distribution Width
SCA: Sickle Cell Anemia
SCD: Sickle cell disease
